# Can we make the basilic vein larger? maneuvers to facilitate ultrasound guided peripheral intravenous access: a prospective cross-sectional study

**DOI:** 10.1186/1865-1380-4-53

**Published:** 2011-08-25

**Authors:** Simon A Mahler, Greta Massey, Liliana Meskill, Hao Wang, Thomas C Arnold

**Affiliations:** 1 Department of Epidemiology and Prevention, Department of Emergency Medicine, Wake Forest University School of Medicine, Winston-Salem, NC, USA; 2 Department of Emergency Medicine, West Virginia University School of Medicine, Morgantown, WV, USA; 3 Department of Anesthesiology, The University of Texas School of Medicine San Antonio, San Antonio, TX, USA; 4 Department of Emergency Medicine, John Peter Smith Health Network, Fort Worth, TX, USA; 5 Department of Emergency Medicine, Louisiana State University Health Sciences Center-Shreveport, Shreveport, LA, USA

## Abstract

**Background:**

Studies have shown that vein size is an important predictor of successful ultrasound-guided vascular access. The objective of this study is to evaluate maneuvers designed to increase basilic vein size, which could be used to facilitate ultrasound-guided peripheral intravenous access (USGPIV) in the Emergency Department (ED) setting.

**Methods:**

This was a prospective non-randomized trial. Healthy volunteers aged 18-65 were enrolled. Basilic veins were identified and the cross-sectional area measured sonographically. Following baseline measurement, the following maneuvers were performed: application of a tourniquet, inflation of a blood pressure (BP) cuff, application of a tourniquet with the arm lowered, and BP cuff inflation with the arm lowered. Following each maneuver there was 30 s of recovery time, and a baseline measurement was repeated to ensure that the vein had returned to baseline. Change in basilic vein size was modeled using mixed model analysis with a Tukey correction for multiple comparisons to determine if significant differences existed between different maneuvers.

**Results:**

Over the 5-month study period, 96 basilic veins were assessed from 52 volunteers. All of the maneuvers resulted in a statistically significant increase in basilic vein size from baseline (*p *< 0.001). BP cuff inflation had the greatest increase in vein size from baseline 17%, 0.87 mm 95% CI (0.70-1.04). BP cuff inflation statistically significantly increased vein size compared to tourniquet placement by 3%, 0.16 mm 95% CI (0.02-0.30).

**Conclusions:**

The largest increase in basilic vein size was due to blood pressure cuff inflation. BP cuff inflation resulted in a statistically significant increase in vein size compared to tourniquet application, but this difference may not be clinically significant.

## Background

Intravenous (IV) access is often required in Emergency Department (ED) patients. Landmark techniques for obtaining peripheral IV access are usually successful, but patients with prior IV drug abuse, obesity, and chronic medical conditions are more likely to have failed attempts [1,2]. Several studies have demonstrated that ultrasound can be used to successfully place peripheral IVs in patients who have failed landmark techniques [1,3-6]. Prior to ultrasound-guided peripheral intravenous access (USGPIV), patients with failed landmark techniques often required central venous cannulation, a procedure with a higher complication rate and demanding more staff resources than peripheral access [2,7].

Studies have shown that vein size is an important predictor of successful ultrasound-guided vascular access [8,9]. While several studies have investigated maneuvers to increase femoral and jugular vein size to facilitate ultrasound-guided central line placement [10-14], few have evaluated maneuvers to increase basilic vein size. Studies evaluating basilic vein size have mainly focused on the creation of an AV fistula for dialysis rather than facilitating USGPIV [15-20]. The objective of this study is to evaluate maneuvers practical for ED use that could be utilized to improve the success of USGPIV by increasing basilic vein size.

## Methods

This was a prospective non-randomized trial, which was approved by the Institutional Review Board of the sponsoring organization. Healthy volunteers aged 18-65 were enrolled over a 5-month period (January to May 2010) at Louisiana State University Health Sciences Center-Shreveport (LSUHSC-S). LSUHSC-S is a tertiary care facility, level one trauma center, and academic center home to a 3-year EM residency program training seven residents per year. Written informed consent was obtained from all volunteers. Volunteers were excluded from the study if they had any acute medical illness or were pregnant.

Volunteers were given a questionnaire to determine if they had undergone venopuncture or vascular access within the previous week, history of upper extremity thrombosis, history of humerus fracture, upper extremity deformity, or upper extremity surgery. If the subjects had any of the above in both arms they were excluded from the study. If the items in the questionnaire were present in only one arm, the volunteer was allowed to participate, but could only use the unaffected arm for the study measurements.

The basilic veins of each subject were identified using a high-frequency linear probe (8-12 MHz, L25 probe on a Sonosite M-Turbo or S-series, Sonosite, Inc., Bothell, WA, USA). After the basilic vein had been identified, two skin marks were made overlying the vein at a point of optimal vein visualization approximately 2-4 cm above the medial epicondyle. If a branching point off the basilic vein was identified within the 2-4 cm area, it was also used as a landmark. The skin marking and branch points were used to ensure that measurements of the vein during different maneuvers occurred at the same location.

**Figure 1:**
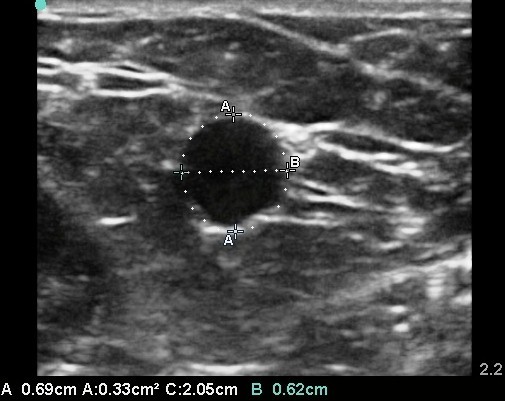
**Ultrasound of basilic vein with measurement of anterior to posterior and medial to lateral diameters**.

Basilic vein measurements for each maneuver were obtained using the following procedures: First, the vein was identified at the location of skin markings on the short axis. Then, the zoom function was used to obtain an enlarged view of the vein, and electronic calipers measured the vein diameter in two dimensions: anterior-posterior and medial-lateral. Using the measurements obtained above, an average vein diameter was calculated. Sonographic measurements were completed by GH and LM, 4th year medical students who had received 1.5 h of didactic and proctored hands-on training in vascular ultrasound prior to the start of this study (see Figure 1).

The first measurement obtained was a baseline measurement in which subjects had their arms supported at the level of the heart. Following baseline measurement, the following maneuvers were performed: application of a tourniquet, inflation of a blood pressure cuff (above diastolic pressure), holding the arm below the level of the heart for more than 30 s prior to the application of a tourniquet, and holding the arm below the level of the heart for more than 30 s prior to inflation of a blood pressure cuff. Measurements of basilic vein size were made before and after each maneuver. Following each maneuver the subject was allowed at least 30 s of recovery time, and a baseline measurement was repeated to ensure that the vein had returned to its normal size (± 0.1 mm).

The effect of each maneuver on vein size was modeled using mixed model analysis. Tukey post hoc analysis was performed to determine if significant differences existed between different maneuvers and adjust for multiple comparisons. Covariance structure was determined by minimizing the AIC (Akaike information criterion), resulting in unstructured covariance. Statistical analysis was preformed with SAS 9.2 (Cary, NC) for Windows.

## Results

Over the 5-month study period from January to May 2010, 96 basilic veins were assessed from 52 volunteers. Of the 52 healthy volunteers, 44 had basilic veins measured in both arms, and 8 subjects had one basilic vein measured. The mean age of the volunteers was 25 (± SD 4 years), 14 (27%) were male, and 38 (73%) were female.

**Figure 2:**
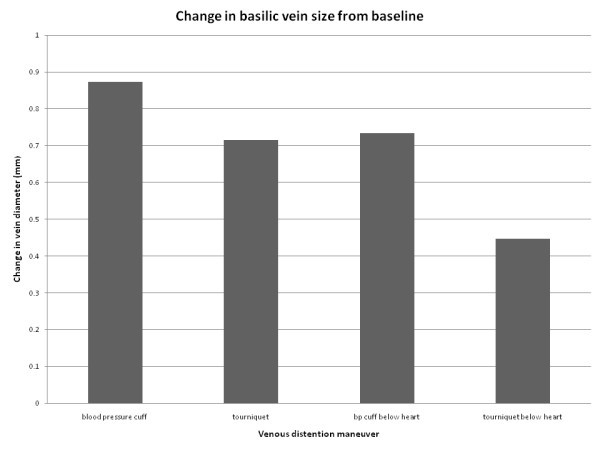
**Difference from baseline basilic vein size (mm) for each manuever**.

The mean baseline diameter of the basilic veins was 5.1 mm (± SD 1.3 mm). Application of a tourniquet with the arm supported at the level of the heart increased size by 14%, a difference of 0.71 mm 95% CI (0.55, 0.88), *p *< 0.001. Inflation of a blood pressure cuff above diastolic pressure, with the arm supported at the level of the heart, increased basilic vein diameter by 17%, 0.87 mm 95% CI (0.70-1.04), *p *< 0.001. BP cuff inflation statistically significantly increased vein size compared to tourniquet placement by 3%, 0.16 mm 95% CI (0.02-0.30), *p *= 0.018 (see Figure 2). All post hoc pairwise comparisons are summarized in Table 1.

**Table 1:** All pairwise comparisons of maneuvers used to dilate the basilic vein.

Maneuver A	Maneuver B	(A-B) Difference in vein diameter (mm)	Adjusted 95% confidence interval of difference (mm)*	Adjusted p*
Baseline	BP cuff	0.87	0.70-1.04	**< 0.0001**
Baseline	Tourniquet	0.71	0.55-0.88	**< 0.0001**
Baseline	BP cuff below heart	0.73	0.52-0.94	**< 0.0001**
Baseline	Tourniquet below heart	0.45	0.25-0.65	**< 0.0001**
Tourniquet below heart	BP cuff	0.43	0.24-0.62	**< 0.0001**
Tourniquet below heart	BP cuff below heart	0.29	0.10-0.48	**0.0006**
Tourniquet below heart	Tourniquet	0.27	0.09-0.44	**0.0005**
Tourniquet	BP cuff	0.16	0.02-0.30	**0.0176**
Tourniquet	BP cuff below heart	0.02	-0.20-0.16	0.9983
BP cuff below heart	BP cuff	0.14	-0.01-0.29	0.0843

Differences measured in mm.

*p values and 95% confidence intervals have been adjusted for multiple comparisons using the Tukey method.

## Discussion

All of the maneuvers tested in our study resulted in a statistically significant increase in basilic vein size. Basilic vein size was increased the most by inflation of a blood pressure cuff above diastolic pressure with the arm supported at the level of the heart. The blood pressure cuff inflated with the arm resting below the heart resulted in the second largest increase in vein size. Blood pressure cuff inflation increased vein size more than a tourniquet or tourniquet applied with the arm below the level of the heart. However, the difference in vein size between BP cuff inflation and tourniquet application was small (3%, 0.16 mm). This difference would result in a change in cross-sectional area of only 5.5% (1.46 mm^2^), which may not be a clinically significant difference for clinicians attempting USGPIV.

Application of a tourniquet with the arm below the level of the heart was the least effective maneuver to increase vein size. On post hoc analysis this maneuver was statistically significantly inferior to all of the other maneuvers. In theory, lowering the arm below the level of the heart for 30 s should have resulted in venous pooling. Therefore, it was expected that the application of a tourniquet in this position would increase basilic vein size compared to a heart level arm postition. It was also expected that lowering the arm before inflation of a blood pressure cuff would increase basilic vein size compared to a blood pressure cuff used at the level of the heart, but this also did not occur. Another study enrolling healthy volunteers and dialysis patients also failed to show a significant difference in cephalic vein size following lowering of the arm and a combination of lowering the arm and warm water emersion [16].

It is unclear why lowering the arm seemed to have a negative impact on basilic vein size compared to the maneuvers performed at heart level. It is possible that despite ensuring that the vein returned to within ± 0.1 mm of its baseline diameter between different maneuvers, recovery time may have been inadequate. Furthermore, volunteers underwent each maneuver in an ordered fashion with maneuvers placing the arm at heart level performed before maneuvers placing the arm below the heart. It is possible that with each maneuver there was some attenuation in the ability of the vein to distend.

Our study differs from prior studies that have examined maneuvers to increase upper extremity vein size, which have mostly evaluated commercial devices or were designed to facilitate vein mapping for dialysis access rather than USGPIV [16,18-20]. Nee et al. investigated antecubital fossa vein size for IV access with the application of a tourniquet versus a tourniquet used in combination with one of two commercially available devices, an Esmarch bandage and a Rhys-Davies exsanguinator. They determined that the combination of either device with a tourniquet was superior to a tourniquet alone [17]. Another study evaluated a vacuum device used with a tourniquet to significantly increase vein size [15]. Other studies have failed to demonstrate significant difference in vein sizes comparing different vein-dilating maneuvers including BP cuff inflation and tourniquets [16,18]. In a study by Planken et al. on patients requiring dialysis access, no significant difference in vein size (cephalic) was found between a tourniquet and a graduated pressure cuff [18]. It is unclear why our results differ from those of Planket et al., but it could be related to differences in the ability to distend veins in dialysis patients compared to healthy volunteers.

Several studies have also investigated maneuvers to increase femoral and jugular vein size to facilitate ultrasound-guided central line placement [10-14]. However, we are not aware of any prior studies investigating maneuvers with the aim of facilitating USGPIV. While USGPIV has a high success rate among patients who have failed landmark techniques, several studies have shown that vein size is an important predictor of successful ultrasound-guided vascular access [1,3-6]. Therefore, maneuvers that can be practically implemented in the ED to increase basilic vein size may improve the success rate of USGPIV [1,8,9].

### Limitations

This study was performed on healthy volunteers, mostly young and female, rather than on patients requiring difficult IV access. Therefore, the results of this study may not be generalizable to patients requiring USGPIV. In addition, sonographic measurements were completed by two relatively inexperienced sonographers, and inter-observer reliability was not assessed. However, prior studies have shown that vein size measurements do not differ significantly between sonographers [18,19].

Temperature changes are known to affect vein size, with warmer temperatures increasing vein size. Warm water emersion has been used as a technique to increased vein size [16,20]. However, our study did not evaluate warm water emersion, because it did not seem practical in the ED setting. While temperature was not directly accounted for in this study, we do not believe that it functioned as a confounder since all of the subjects served as their own controls. Volunteers were present in the same climate-controlled environment throughout their exposure to the different maneuvers.

As previously mentioned, the decreased effectiveness of maneuvers completed with the arm resting below the level of the heart may have been the result of bias. Although procedures were utilized to ensure that the basilic vein returned to baseline size between different maneuvers, it is possible that our results were biased by inadequate recovery time. Also, sequence bias may have occurred, as the ability of veins to dilate may have been attenuated over time or with repetitive maneuvers. Future studies should have longer recovery periods and vary the sequence of the maneuvers studied.

In addition, some of the differences between maneuvers, while statistically significant, were small and may not be clinically significant. Furthermore, although prior studies have demonstrated that larger vein size improves USGPIV success, the subjects in this study were volunteers and did not have USGPIV performed. Therefore, further study is required to determine if specific maneuvers used for venous distention increase USGPIV success relative to other maneuvers.

## Conclusions

All of the maneuvers tested resulted in a statistically significant increase in basilic vein size. Inflation of a blood pressure above diastolic pressure with the arm supported at the level of the heart produced the largest increase in basilic vein size. BP cuff inflation resulted in a statistically significant increase in vein size compared to tourniquet application, but this difference may not be clinically significant. The least effective maneuver was the application of a tourniquet with the arm resting below the level of the heart. Future investigation of these maneuvers designed to facilitate USGPIV should study patients with failed landmark IV techniques, have long recovery periods between maneuvers, and vary the sequence of the maneuvers studied.

## Patient Consent

Written informed consent was obtained from all study volunteers.

## Competing interests

The authors declare that they have no competing interests.

## Authors' contributions

SM was involved in the study design, statistical analysis, and manuscript preparation. GM and LM were involved in the study design and carrying out study measurements. HW provided statistical support and was involved in the study design. TA was involved in manuscript preparation. All authors read and approved the final manuscript.
